# Physical activity, confidence and quality of life among cancer patient-carer dyads

**DOI:** 10.1186/s40798-021-00333-7

**Published:** 2021-07-01

**Authors:** Siu-man Ng, Melody H. Y. Fung, Jessie S. M. Chan, Celia H. Y. Chan, Cecilia L. W. Chan

**Affiliations:** 1 Department of Social Work and Social Administration, The University of Hong Kong, 5/F, Jockey Club Tower, The Centennial Campus, Pokfulam, Hong Kong, China; 2 Centre on Behavioral Health, The University of Hong Kong, Pokfulam, Hong Kong, China; 3 School of Chinese Medicine, Faculty of Medicine, The University of Hong Kong, Pokfulam, Hong Kong, China

**Keywords:** Mixed cancer, Patient-caregiver dyads, Physical activity, Quality of life, Mediation, Path analysis

## Abstract

**Background:**

Physical activity (PA) has been positively associated with health-related quality of life (HRQoL) among cancer patients and family caregivers. However, there has been no relevant research for patient-caregiver dyads.

**Methods:**

Path analysis, based on the actor–partner interdependence model (APIM), was used to examine the relationship between physical activity and health-related quality of life and explore the mediating role of emotional distress in 233 dyads.

**Results:**

In both patients and caregivers, physical activity had a direct positive effect on physical quality of life (QoL) but not on mental. There was a significant indirect effect of physical activity on health-related quality of life via emotional distress for both dyad members. Patients’ and caregivers’ confidence in fighting cancer was negatively associated with their own emotional distress. Caregivers’ confidence in fighting cancer was positively associated with their physical activity and also negatively associated with patients’ emotional distress.

**Conclusions:**

Physical activity may be considered as a possible behavioral and rehabilitation strategy for improving health-related quality of life in patient-caregiver dyads and reducing negative symptoms. Future research and intervention may consider cancer patient-family caregiver dyad as a unit of care.

## Key point

- Limited research has addressed the physical activity and health-related quality of life of patient-family caregiver dyads.
- Physical activity could potentially serve as behavioral and rehabilitation strategy for improving health-related quality of life in patient-caregiver dyads and reducing negative symptoms.
- Patient-family caregiver dyad may be considered as a unit of care in future research and intervention.

## Background

Cancer has an adverse effect on health-related quality of life (HRQoL) in patients [1] and their caregivers [2]. Previous studies have associated poor HRQoL with higher levels of stress [3, 4], anxiety, and depression [5, 6], while physical activity (PA) provides physiological and psychological benefits [7], including reductions in symptoms of depression [8] and emotional distress [9] and improvements in cancer patients’ HRQoL during active treatment and rehabilitation [6, 7, 10, 11]. A recent review suggested that exercise should be used as an adjunct to standard care [12]. The Clinical Oncology Society of Australia (COSA) also acknowledges the role of physical activity in counteracting adverse effects of cancer and its treatment [13].

Family caregivers have a higher risk of stress, depression, poorer physical health [14], lower psychosocial HRQoL, poorer general health [15], and even higher mortality [16] as compared with the general population. Caregiving may interfere with caregivers’ lifestyle and health-promoting behaviors which may result in their insufficient PA [4]. Previous studies have demonstrated lower levels of PA among caregivers than non-caregivers, and those with lower PA levels have more depressive symptoms [17] and worse psychological health [15]. Limited yet important research on PA among family caregivers [2, 18] has shown that PA improves caregivers’ quality of life (QoL) [2] and reduces their stress, depression, and burden [18].

A meta-analysis concluded interdependence between patients and their caregivers’ experiences of psychological distress; family caregivers are negatively affected by the patients’ cancer disease while their behavior can in turn influence patient outcomes due to the closeness of the relationship [19]. Reciprocity of emotional well-being has been found among patient-caregiver dyads [20], which reflects the importance of mutual support between the caregivers and patients in the recovery process. In addition, caregivers play an important role increasing patients’ PA because they act as role models to inspire and increase patients’ self-efficacy with respect to PA [21]. Facilitating regular PA among caregivers is therefore beneficial to both caregivers and patients’ well-being [5]. Given the interdependence of emotional well-being as suggested by previous findings, it might be more efficient to deliver information and care to patients-caregiver dyad as a unit instead of intervening individually,

Previous research has led to a model which predicts HRQoL of both cancer patients and their caregivers based on demographic, clinical, and psychosocial characteristics [22, 23]. These variables include gender, age, socioeconomic status, religious activity, time since diagnosis, therapy status, and confidence in fighting cancer. Other research also demonstrated that PA improved HRQoL of cancer patients [6, 7, 10, 11] and their caregivers [2]; however, there has been no research into the relationships between antecedent factors (person and disease treatment), PA, and HRQoL in cancer patients and their caregivers. While a study of the associations between cancer coping styles and health-related behaviors found that cancer patients with greater “fighting spirit” in their coping reported higher levels of PA [24], it is still unclear whether patients and caregivers’ confidence in fighting cancer may also influence their level of PA.

**Fig. 1:**
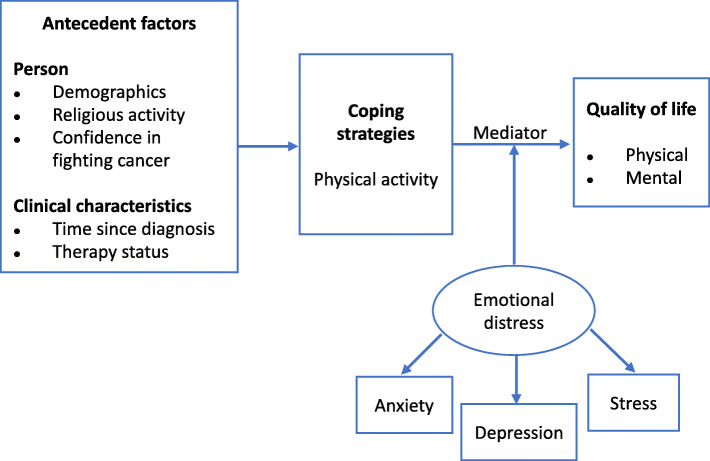
Conceptual model

The aim of this study was to investigate the relationship between PA and HRQoL in patient-caregiver dyads through path analysis. We used cross-sectional dyadic data and a modification of the stress-coping model [22]; we assumed that a set of antecedent factors including demographic and clinical characteristics were associated with PA as a coping strategy and influenced HRQoL as the outcome (see Fig. 1). In particular, we hypothesized that PA was both directly and indirectly associated with HRQoL through emotional distress as a potential mediator [10].

## Methods

### Design and procedures

Here we report baseline data from a randomized controlled trial that examined the effects of Qigong exercise on health-related outcomes in Hong Kong, in people with mixed cancer and their caregivers. We recruited participants through adverts in the mass media. The inclusion criteria were (1) caregiver had no current diagnosis of cancer, (2) patient at their post-treatment stage with screened physical ability to stand for 30 min of physical exercise, and (3) patient and caregiver had to participate as a pair. The exclusion criteria for both dyad members were as follows: (1) physical impairment which restricted body movement, (2) cognitive impairment which prevented understanding of instructions, (3) comorbid major psychiatric illness other than depression or anxiety, and (4) pregnancy. Two hundred and thirty-eight patient-caregiver dyads gave informed consents and completed the questionnaires. We excluded five pairs because of problems with completed questionnaires (incorrect age data *n* = 1; missing caregiver data *n* = 2; missing patient data *n* = 2) yielding a final sample of 233 patient-caregiver dyads. All of the procedures were approved by the institutional review board of the University of Hong Kong.

### Measures

Physical activity was assessed using the International Physical Activity Questionnaire - short form [25]. This nine-item instrument captures self-reported physical activity in the week before completion. Responses are converted to Metabolic Equivalent Task minutes per week (MET-min/week) by multiplying the total number of minutes spent in vigorous activity, moderate intensity activity, and walking over the week by 8.0, 4.0, and 3.3, respectively. The MET scores for each activity level are summed to give an indication of overall physical activity.

Emotional distress was assessed as a latent construct using validated measures of perceived stress, anxiety, and depression as indicators. Perceived stress was measured using the ten-item Chinese Perceived Stress Scale [26]. Responses are given using a five-point scale, total scores range from 0 to 40 and high scores indicate high stress levels. Anxiety and depression were measured using the Chinese Hospital Anxiety and Depression Scale (HADS) [27], a fourteen-item instrument that captures the severity of anxiety and depressive symptoms using a four-point response scale. Total scores for anxiety (7 items) and depression (7 items) range from 0 to 21, higher scores denote more severe symptoms.

QoL was assessed using the Chinese short-form (SF)-12 questionnaire [28, 29]. This twelve-item health survey measures physical and mental QoL using separate 0–100 subscales, higher scores indicate better QoL. In this study, all measurement scales had satisfactory reliability with Cronbach’s α exceeding .70 in both patient and caregiver samples.

We used a single item to assess patients’ and caregivers’ confidence in fighting cancer. The item was self-constructed by the research team.

### Data analysis

Path analysis was conducted with Mplus version 7.11 [30] using maximum likelihood estimation. Missing data were handled with full information maximum likelihood [31] under the missing at random assumption. Analysis of dyadic data is relatively complex because of the dependence that arises from shared characteristics and experiences; failure to account for this would result in underestimation of the standard errors and bias the results. We used the Actor-Partner Interdependence Model (APIM) [32] to handle interdependence. The APIM model models the responses of both dyad members simultaneously and allows variable interactions between them whilst specifying correlations for all pairs of variables across the dyad. This model not only estimated the conventional actor effects for both the patients and caregivers, but also patient-partner effects (patient predictors influencing caregiver outcomes) and caregiver-partner effects (caregiver predictors influencing patient outcomes).

The proposed path model included covariates (gender, age, socioeconomic status, religious activity, time since diagnosis, therapy status, and confidence in fighting cancer), PA as the predictor, emotional distress as the latent mediator, and physical and mental QoL as outcome variables. We evaluated the direct effect of PA on QoL and the indirect effect of PA on QoL via emotional distress. Model fit was assessed using the following fit indices [33]: comparative fit index (CFI) ≥ .95; Tucker-Lewis index (TLI) ≥ .95; root mean square error of approximation (RMSEA) ≤ .06 and 90% confidence interval (C.I.); and standardized root mean square residual (SRMR) ≤ .06. To allow for potential non-normal distribution of the indirect effects, bootstrapping with 5000 re-samples was used to produce bootstrap asymmetric 95% CI [34]. *R* squared values were computed for the study variables to determine the amount of variance accounted for by the model.

## Results

### Participants and descriptive statistics

The patient sample consisted of cancer patients who had been diagnosed with lung cancer (24.0%), breast cancer (19.3%), colorectal cancer (14.2%), female reproductive cancers (cervical cancer, ovarian cancer, or uterine cancer; 7.3%), nasopharyngeal cancer (6.4%), esophageal and stomach cancer (3.0%), prostate cancer (2.6%), non-Hodgkin’s lymphoma (2.6%), thyroid cancer (1.7%), and other types of cancer (18.0%). Table 1 shows the demographic profile of the 233 patient-caregiver dyads. Both samples had a similar demographic profile, with the majority being female, married, and of high socioeconomic status. Accommodation is taken as a rough indicator of socioeconomic status in Hong Kong [35]. Participants who own or rent a private housing are supposed to be relatively wealthy and therefore classified as having high socioeconomic status. Spouses made up the majority of caregiver (62.7%).

**Table 1:** Demographic profile for patients and caregivers

*N* = 233	Patients	Carers
	*N* (%)	*N* (%)
Gender		
*Female*	138 (59.2)	146 (62.7)
Education level		
*Primary or less*	57 (24.6)	37 (16.0)
*Secondary*	107 (46.3)	106 (45.9)
*Tertiary*	67 (29.0)	88 (38.1)
Marital status		
*Married*	194 (83.3)	198 (85.0)
Religious activity		
*Yes*	119 (51.1)	104 (44.6)
Socioeconomic status		
*High*	122 (52.4)	125 (53.6)
Caregiver’s relationship to cancer patient		
Spouse	146 (62.7%)	
Parent	11 (4.7%)	
Child	31 (13.3%)	
Others	45 (19.3%)	
	*M (SD)* *Median (interquartile)*	*M (SD)*
Age (years)	57.4 (10.4)	53.6 (12.7)
Time since diagnosis (years)	*1.0 (1.0 – 2.0)*	/
Cancer stage		
0	8 (3.4%)	
1	43 (18.5%)	
2	39 (16.7%)	
3	70 (30.0%)	
4	73 (31.3%)	
Therapy status		
Completed	92 (39.5%)	
In therapy	110 (47.2%)	
Waiting for therapy	31 (13.3%)	
Number of therapies	2.0 (1.1)	/
Confidence in fighting cancer	3.5 (1.0)	3.5 (1.0)

The mean ages of patients and caregivers were 57.4 years (*SD* = 10.4) and 53.6 years (*SD* = 12.7), respectively. The most common cancer stage in patients was stage 4 (31.3%), followed by stage 3 (30.0%), stage 1 (18.5%), stage 2 (16.7%), and finally stage 0 (3.4%). The majority (60.5%) of the patients were in therapy or waiting for therapy. The median time since diagnosis was 1 year (interquartile range: 1–2 years) and patients were receiving an average 2.0 (*SD* = 1.1) types of therapy at the time of the study. Confidence in fighting cancer was expressed using a five-point scale ranging from 1 (not at all confident) to 5 (very confident), and the mean score for both patients and caregivers was 3.5 (*SD* = 1.0).

Table 2 displays the descriptive statistics for study variables; the scores of members of a dyad were positively correlated with respect to PA, perceived stress, anxiety and depression, and mental QoL but not physical QoL. The patients reported higher perceived stress, anxiety, and depression and lower PA and physical and mental QoL than the caregivers. Using a threshold score of 8 [36], 61.8% and 56.2% of the patients were classified as potentially suffering from clinically significant anxiety and depression, respectively. In caregivers, the corresponding figures were 51.1% and 37.8% for anxiety and depression, respectively. Table 3 shows the bivariate associations between PA and other study variables separately for cancer patients and family caregivers.

**Table 2:** Descriptive statistics of study variables for patients and caregivers (*N* = 233)

	Patients	Carers		
	*M (SD)*	*M (SD)*	*t value*	*Correlation coefficient r*
Physical activity (ln)	7.3 (1.1)	7.6 (1.2)	3.49**	0.156*
Perceived stress	20.6 (4.8)	19.7 (5.0)	2.47*	0.385**
Anxiety	8.8 (4.1)	7.6 (3.7)	4.47**	0.407**
Depression	8.4 (4.3)	6.2 (3.8)	7.83**	0.424**
Physical quality of life	37.2 (8.2)	45.2 (8.7)	10.7**	0.088
Mental quality of life	41.6 (10.3)	45.4 (9.8)	4.78**	0.249**

*t* test for comparison between cancer patients and caregivers

*p < 0.05; **p < 0.01

**Table 3:** Correlation coefficients among measures on physical activity, emotional distress, and quality of life for cancer patients and caregivers

For cancer patients					
	PA	PSS	Anxiety	Depression	P-QoL
PA	1				
PSS	− .096	1			
Anxiety	− .173*	.740***	1		
Depression	− .172*	.632***	.784***	1	
P-QoL	.226**	− .335***	− .355***	− .465***	1
M-QoL	.217**	− .579***	− .736***	− .727***	.215**
For family caregivers					
	PA	PSS	Anxiety	Depression	P-QoL
PA	1				
PSS	− .090	1			
Anxiety	− .158*	.723***	1		
Depression	− .188**	.590***	.746***	1	
P-QoL	.176**	− .264***	− .293***	− .424***	1
M-QoL	.157*	− .659***	− .685***	− .657***	.190**

### Path model results

**Fig. 2:**
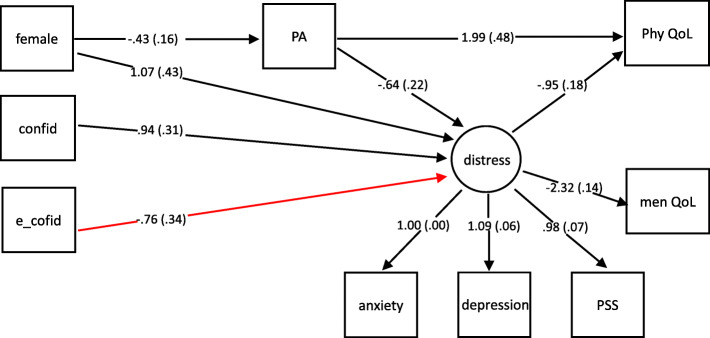
Path model on the relationships between PA and QoL via emotional distress for patients. Note: All paths coefficients shown are significant with *p* < .05 and refer to actor effects, except the partner effect from caregivers’ confidence in fighting cancer to patients’ emotional distress (red and bolded arrow); confid = confidence in fighting cancer; PA = physical activity; PSS = perceived stress; phy QoL = physical quality of life; men QoL = mental quality of life

**Fig. 3:**
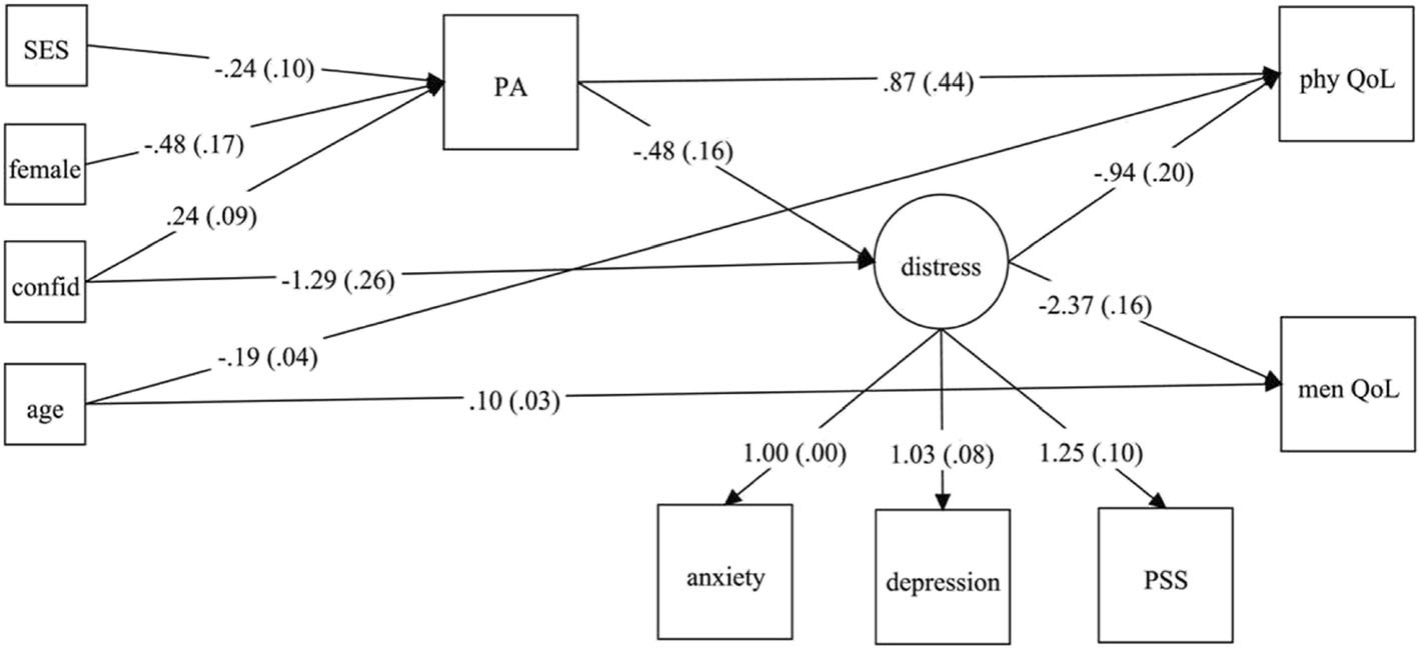
Path model on the relationships between PA and QoL via emotional distress for caregivers. Note: All paths coefficients shown are significant with *p* < .05 and refer to actor effects; confid = confidence in fighting cancer; SES = socioeconomic status; PA = physical activity; PSS = perceived stress; phy QoL = physical quality of life; men QoL = mental quality of life

The path model displayed an acceptable fit to the data (CFI = .97; TLI = .96; RMSEA = .036, 90% CI: .020–.049; SRMR = .045). Detailed results are presented for patients and caregivers in Figs. 2 and 3, respectively. The model explained 6.6% and 11.4% of the variance in PA in patients and their caregivers, respectively. Women reported lower levels of PA both as patients (*β* = − 0.43, *p <* .01) and as caregivers (*β* = − 0.48, *p <* .01). In caregivers, PA was positively associated with confidence in fighting cancer (*β* = 0.24, *p <* .05). Caregivers with high socioeconomic status reported lower level of PA (*β* = − 0.24, *p <* .05).

#### Patients’ quality of life

The model explained 29.5% of the variance in patients’ physical QoL and 65.9% of the variance in their mental QoL. In patients, PA had a direct positive effect on physical QoL (*β* = 1.99, *p <* .01) but not on mental QoL (*β* = 0.42, *p =* .34). Emotional distress had negative effects on both physical QoL (*β* = − 0.95, *p <* .01) and mental QoL (*β* = − 2.32, *p <* .01). PA had indirect positive effects on physical QoL (αβ = 0.61, *p* < .01) and mental QoL (αβ = 1.48, *p* < .01) via emotional distress. In patients, PA was positively linked with QoL via several steps including a negative relationship between PA and emotional distress, and then a relationship between emotional distress and physical/mental QoL, suggesting that patients with higher level of PA had lower level of emotional distress, which led to higher levels of QoL.

#### Patients’ emotional distress

The model explained 27.1% of the variance in patients’ emotional distress. PA had a direct negative effect on emotional distress (*β* = − 0.64, *p <* .01). Higher levels of emotional distress were reported by female patients (*β* = 1.07, *p <* .05), patients with less confidence in fighting cancer (*β* = − 0.94, *p <* .01), and patients who had caregivers with less confidence in fighting cancer (*β* = − 0.76, *p <* .05).

#### Caregivers’ quality of life

The model explained 24.2% of the variance in caregivers’ physical QoL and 60.6% of the variance in their mental QoL. In caregivers, PA had a direct positive effect on physical QoL (*β* = 0.87, *p <* .05) but not on mental QoL (*β* = − 0.15, *p =* .68). Emotional distress had negative effects on both physical QoL (*β* = − 0.94, *p <* .01) and mental QoL (*β* = − 2.37, *p <* .01). PA had indirect effects on physical QoL (αβ = 0.45, *p* < .01) and mental QoL (αβ = 1.14, *p* < .01) via emotional distress. Older caregivers reported lower physical QoL (*β* = − 0.19, *p <* .01) but better mental QoL (*β* = 0.10, *p <* .01).

#### Caregivers’ emotional distress

The model explained 21.5% of the variance in caregivers’ emotional distress. PA had a negative effect on caregivers’ emotional distress (*β* = − 0.48, *p <* .01). Caregivers with less confidence in fighting cancer (*β* = − 1.29, *p <* .01) reported higher emotional distress.

#### Mediating effects

In cancer patients, emotional distress mediated the association between their confidence in fighting cancer and their physical QoL (αβ = 0.89, bootstrap 95% CI: 0.36–1.65, *p* < .01). The patient’s emotional distress also mediated the relationship between a caregiver’ confidence in fighting cancer and the patient’s physical QoL (αβ = 0.72, bootstrap 95% CI: 0.10–1.54, *p* < .05). Patients’ emotional distress mediated the association between their confidence in fighting cancer and their mental QoL (αβ = 2.18, bootstrap 95% CI: 0.86–3.61, *p* < .01). The patient’s emotional distress also mediated the relationship between a caregiver’s confidence in fighting cancer and the patient’s mental QoL (αβ = 1.76, bootstrap 95% CI: 0.16–3.28, *p* < .05).

Caregivers’ confidence in fighting cancer had indirect effects on their physical QoL via PA (αβ = 0.21, bootstrap 95% CI: 0.02–0.55, *p* < .05), via emotional distress (αβ = 1.21, bootstrap 95% CI: 0.75–1.87, *p* < .01), and via PA and emotional distress (αβγ = 0.11, bootstrap 95% CI: 0.02–0.29, *p* < .01). Similarly, caregivers’ confidence in fighting cancer had indirect effects on their mental QoL via emotional distress (αβ = 3.04, bootstrap 95% CI: 1.94–4.26, *p* < .01) and via PA and emotional distress (αβγ = 0.27, bootstrap 95% CI: 0.06–0.65, *p* < .01).

## Discussion

To the best of our knowledge, this is the first study to examine how PA is related to QoL in cancer patient-caregiver dyads. We developed a path model of the associations between PA and HRQoL that indicated positive correlations between the members of a dyad with respect to mental QoL, PA, perceived stress, anxiety, and depression. These findings are in line with results of Kershaw et al. [21] regarding positive correlations between the members of a dyad with respect to mental QoL, but not physical QoL in a sample of 121 prostate cancer patients-spouse dyads. These results suggest that clinicians should consider the care requirements of both members of the cancer patient-family caregiver dyad [18].

Our study demonstrated that PA has direct or indirect positive effects on HRQoL for both cancer patients and family caregivers, which is consistent with previous systematic reviews [7, 11, 37]. However, few studies have investigated the role of PA in determining the HRQoL of the family caregivers of cancer patients [18]. This study has showed that PA was positively associated with physical and mental QoL in cancer patient-family caregiver dyads. Unlike findings from a previous population-based study among Iranian adult population which indicated higher level of PA among people with high SES [38], our findings indicate association of caregivers with high SES with low PA. Further research may be conducted to investigate barriers or concerns among caregivers with high SES in performing PA.

Among patients, PA had direct effects on emotional distress and physical QoL but not on mental QoL. Physical QoL was positively associated with PA, while both physical and mental Qol were negatively associated with emotional distress. Emotional distress mediated the association between PA and physical QoL. The results are in line with findings of a large cohort study of colorectal cancer survivors (*n* = 1371) using multiple linear regression [10]. In our sample, the relationship between PA and mental QoL was also entirely indirect, whereas the relationship between PA and physical QoL was both direct and indirect, which is consistent with the findings of a previous study of patients with multiple sclerosis [39].

In family caregivers, PA directly influenced physical QoL and emotional distress and indirectly affected mental QoL via emotional distress, which is in line with a review of PA intervention on stress and depression among caregivers of people with dementia [18]. Emotional distress was also found to mediate the association between PA and physical QoL. Their age was negatively associated with physical QoL and positively associated with mental QoL, which are consistent with previous research [22].

We assessed confidence in fighting cancer in both cancer patients and their family caregivers; it can be considered a similar construct to the form of self-efficacy used in other studies [22]. Self-efficacy of cancer patients is defined in terms of confidence in managing stress and the changes associated with cancer or treatment, whereas that of family caregivers is defined in terms of confidence in managing the cancer as a caregiver. In this study, confidence in fighting cancer was positively associated with PA in caregivers but not patients. It was negatively associated with emotional distress in both cancer patients and family caregivers. The finding is consistent with a previous study of a relatively small sample of colorectal cancer survivors (*n* = 62) and their family caregivers (*n* = 42) [40]. We also found that confidence in fighting cancer had indirect positive effects on HRQoL via emotional distress in the dyads. These results suggest that confidence in fighting cancer or self-efficacy should be assessed as part of the clinical evaluation of both patients and caregivers [22]. Both PA and confidence in fighting cancer/self-efficacy could be important targets for behavioral and self-management interventions aimed at improving HRQoL [41]. A previous study suggested that family caregivers’ social support is essential to cancer patients’ participation in PA [21]. Interestingly, although we did not find an association between a caregiver’s confidence in fighting cancer and the patient’s PA, we found that family caregiver’s confidence in fighting cancer was negatively associated with the cancer patient’s emotional distress. This implies that a family caregiver’s confidence in fighting cancer plays an important role in determining a cancer patient’s emotional distress and HRQoL, supporting the argument that family caregivers should be involved in intervention designed to reduce cancer patients’ emotional distress and improve their HRQoL [19].

### Implications

This study has a number of clinical implications. First, the positive association between PA and HRQoL in cancer patient-family caregiver dyads suggests that healthcare professionals should use PA as part of interventions aimed at improving the HRQoL of such dyads. Results also suggest that healthcare providers should consider screening individuals to identify those with low confidence in fighting cancer.

Levels of PA were lower in female patients and caregivers than in their male counterparts, which is consistent with recent studies of students and colorectal cancer patients [42]. These results suggest that women need more encouragement to engage in PA when they are cancer patients or caregivers for a patient with cancer. In line with previous research [19], female cancer patients were more likely to report significant emotional distress than male cancer patients. Furthermore, the physical condition of patients with various types of cancer may differ and thus affect their ability and confidence of performing PA. Patients who need additional assistance on recovery from surgeries and their caregivers who have to pay extra effort in taking care of the patients may be more reluctant to spend time on doing PA. Healthcare providers could apply findings of this study to identify high-risk individuals in terms of PA and confidence in fighting cancer.

### Limitations

First, the self-selection recruitment method may have resulted in selection bias. These findings may not generalize to patients whose spouse or other family caregiver is not willing to participate in research of this kind. Second, the sample was predominantly female, well-educated, and married. It is important to determine whether the model presented here is applicable to cancer patients with a different demographic profile. Third, the use of self-report measures was subject to common method bias, which may have inflated correlations among the study variables. Future studies could assess the effects of PA on objectively measurable psychophysiological outcomes. Fourth, we relied on cross-sectional data, which precludes inferences about the causal relationships among the variables. Longitudinal research investigating the impact of changes in PA and emotional distress on HRQoL is needed in the future. Fifth, confidence in fighting cancer was assessed using a single question devised by the research team; this indicator was shown to be associated with PA in family caregivers and with emotional distress in both cancer patients and family caregivers. In future research, it would be advisable to use a validated scale for measuring self-efficacy, self-esteem, or sense of hopelessness instead.

## Conclusions

This study is the first to investigate the role of PA in determining HRQoL in a mixed sample of 233 cancer patient-family caregiver dyads. Notwithstanding the limitations, it shows the importance of PA and confidence in fighting cancer to HRQoL in such dyads and supports the development of activity or exercise interventions as a strategy for improving HRQoL in patients and caregivers throughout the course of the disease. It also suggests how healthcare providers could identify individuals who likely to have low levels of PA in order to provide appropriate interventions.

## Data Availability

The datasets used and analysed during the current study are available from the corresponding author on reasonable request.

## Abbreviations

PA: Physical activity
HRQoL: Health-related Quality of Life
APIM: Actor–partner interdependence model
QoL: Quality of life
